# Targeting endoplasmic reticulum stress-induced CLGN resensitizes hepatocellular carcinoma to apoptosis: paeonol synergistically enhances efficacy by dual inhibition of CLGN and NF-κB

**DOI:** 10.3389/fonc.2025.1709962

**Published:** 2025-11-28

**Authors:** Sitong Yan, Anqi Wang, Xiang Chen, Weijia Jiang, Xiao Du, Yuhan Huang, Xiangyu Zu, Yue Zhu, Jiatao Liu, Lulu Fan, Lingling Zhang, Guoping Sun

**Affiliations:** 1 Department of Integrated Traditional Chinese and Western Medicine, The First Affiliated Hospital of Anhui Medical University, Hefei, Anhui, China; 2 Department of Oncology, The First Affiliated Hospital of Anhui Medical University, Hefei, Anhui, China; 3 Department of Oncology, The First Affiliated Hospital of Nanchang University, Nanchang, China; 4 Department of Oncology, The First Affiliated Hospital of Bengbu Medical University, Bengbu, Anhui, China; 5 Department of Pharmacy, The First Affiliated Hospital of Anhui Medical University, Hefei, Anhui, China; 6 Institute of Clinical Pharmacology, Anhui Medical University, Hefei, Anhui, China

**Keywords:** hepatocellular carcinoma, paeonol, CLGN, endoplasmic reticulum stress, combination therapy

## Abstract

**Purpose:**

Endoplasmic reticulum stress (ERS) drives hepatocellular carcinoma (HCC) progression and therapy resistance. This study identifies Calmegin (CLGN) as a novel ERS-induced pro-survival factor and explores shCLGN combined with Paeonol (Pae) to overcome apoptosis resistance via NF-κB suppression.

**Materials and methods:**

CLGN was discovered by transcriptome sequencing of tunicamycin (TM)-induced ERS in HepG2 cells and validated via Western blot. Clinical significance was assessed using 93 paired HCC/adjacent tissues (IHC/WB) and TCGA data. Functional roles of CLGN (proliferation: CCK-8/EdU; migration/invasion: Transwell; apoptosis: flow cytometry). shCLGN efficacy alone or with Pae was tested *in vitro* and *in vivo* (xenografts). Transcriptome sequencing combined with Western blot was performed after sh-CLGN to confirm that CLGN regulates apoptosis-related proteins (such as Bcl-2, Bax, and Caspase-3) through the NF-κB pathway, as well as the mechanism of sh-CLGN combined with paeonol on the pathway.

**Results:**

ERS significantly upregulated CLGN in HCC, correlating with advanced tumor stage and poor prognosis. CLGN promoted proliferation/migration and suppressed apoptosis. Crucially, sh-CLGN sensitized HCC cells to Pae, synergistically enhancing apoptosis and tumor suppression. Mechanistically, CLGN sustained survival via NF-κB activation; the combination (sh-CLGN + Pae) dual-blocked CLGN/NF-κB, reversing pro-survival signaling *in vitro* and *in vivo*.

**Conclusion:**

CLGN is a pivotal ERS effector mediating HCC apoptosis resistance through NF-κB. Sh-CLGN combined with Pae restores apoptotic sensitivity via dual CLGN/NF-κB inhibition, providing a potent strategy against ERS-adapted HCC.

## 1 Introduction

Hepatocellular carcinoma (HCC) ranks among the most prevalent cancers worldwide (1, 2). According to the 2022 Global Cancer Statistics, liver cancer exhibits the third highest mortality rate among all cancers, accounting for 7.8% of total cancer-related deaths (3). Systemic therapy is crucial for patients with advanced liver cancer, but current treatments are limited due to high drug resistance (4). Therefore, novel therapeutic strategies are urgently needed (5).

HCC progression and therapy resistance are driven by a complex network of molecular alterations. These include classic genetic abnormalities (e.g., TERT, TP53, CTNNB1) and dysregulated signaling pathways (e.g.,Wnt/β-catenin, PI3K/AKT/mTOR) (6–8), as well as non-canonical pathways such as Hippo/YAP, NF-κB, and STAT3 (9). ERS-induced unfolded protein response (UPR) promotes tumor progression and confers drug resistance. For instance, extracellular vesicles from selenium-deficient cells propagate ERS signals via the PI3K-AKT-mTOR axis (10), and the COPI-MAP2K3 pathway provides a novel link between ERS and YAP activation (11).

At its core, ERS triggers the UPR through three key sensors—PERK, ATF6, and IRE1α (5, 12)—reprogramming tumor cell metabolism and driving malignant progression (13)Building on this foundation, our research has identified specific ERS-mediated mechanisms in HCC (14, 15). We discovered that an ERS-linked super-enhancer promotes epithelial-mesenchymal transition (EMT) by activating TNC through CREB5 (16) Furthermore, we demonstrated that the ARHGEF2/EDN1 pathway contributes to ERS-related drug resistance by promoting angiogenesis and proliferation (17), and that NAT10 enhances metastasis and lenvatinib resistance by stabilizing HSP90AA1 mRNA via ac4C modification (18).

The CLGN gene encodes the endoplasmic reticulum-associated protein Calmegin (19). As a chaperoneprotein of the endoplasmic reticulum, CLGN plays a significant role in protein folding, cellular stress responses, and calcium ion homeostasis, influencing both the physiological and pathological processes of cells (19). For instance, in Kidney Renal Clear Cell Carcinoma (KIRC), CLGN has been identified as a prognostic marker associated with necroptosis (20). These findings not only offer a new perspective on the role of CLGN in diseases but also lay the groundwork for its exploration as a potential therapeutic target. However, the molecular mechanisms underlying its pro-oncogenic and drug-resistant effects in ERS responses in hepatocellular carcinoma remain unexplored systematically. This research gap highlights the scientific value and clinical translational potential of further investigating the interplay between CLGN and HCC under ERS conditions.

Traditional Chinese medicine has been widely employed in China owing to its favorable safety profile, rapid efficacy, and low cost. As documented in the Chinese Pharmacopoeia, Moutan Cortex (the root bark of Paeonia suffruticosa) (21)is characterized by a bitter and pungent taste, slightly cold nature, and tropism toward the heart, liver, and kidney meridians (22, 23). According to “Dian Nan Ben Cao,” Moutan Cortex breaks blood, promotes blood circulation, treats masses, and eliminates heat from the blood (24). Paeonol (Pae), a phenolic compound derived from Moutan Cortex, exhibits diverse biological activities including anti-inflammatory, antitumor, and cardioprotective effects (21, 25). Although Pae has shown antitumor activity and enhanced chemotherapeutic efficacy in various cell lines and animal models (26), its mechanism of action in HCC under ERS conditions remains unreported.

In this study, we analyzed transcriptome sequencing data and reported significantly increased CLGN expression levels in HCC, which is associated with ER stress. Furthermore, inhibition of CLGN expression reversed ERS-induced resistance to Pae, suggesting a promising combinatory therapeutic strategy.

## 2 Materials and methods

### 2.1 Clinical sample collection

In all, 93 pairs of clinical samples, which included HCC tissues in addition to adjacent nontumor tissues, were procured from the First Affiliated Hospital of Anhui Medical University (NO.20040158). Informed consent was obtained from all participants in the study; the research protocol was approved by the Ethics Committee of the First Affiliated Hospital of Anhui Medical University and was in compliance with the Helsinki Declaration guidelines. Patient-specific data were meticulously recorded in accordance with the tumor–node–metastasis (TNM) classification system as defined by the Union for International Cancer Control (UICC) (27). Furthermore, we acquired 8 pairs of matched HCC tissues and adjacent normal tissues (ANTs) for Western blot analysis.

### 2.2 Immunohistochemistry

Tissue microarrays consisting of 93 pairs of HCC/ANT samples were constructed. Immunohistochemical staining was performed via a two-step method with a human monoclonal anti-rabbit CLGN antibody (1:100, BOSTER), a KI-67 antibody (1:400, CST), and an NF-κB antibody (1:400, CST).

The positive signal intensity scores were as follows: (negative, 0; weak, 1; moderate, 2; or strong, 3). The percent positivity was determined by evaluating the proportion of positive cells and was categorized as follows: (<5%, 0; 5%–25%, 1;26%–50%, 2; 51%–75%, 3; 76%–100%, 4). The staining index (SI) was calculated as follows: SI = signal intensity × proportion of positive cells (28). The SI ranged from 0–12, with a median of 6. Using an SI value of 6 as the threshold, samples were classified as either low-expression (SI < 6) or high-expression (SI > 6).

### 2.3 Cell culture

Hep-G2, Huh-7, MHCC97H, LM3, and Hep-3B cells were obtained from the Chinese Academy of Sciences (Shanghai). The cell culture medium contained 10% fetal bovine serum (ExCell,Suzhou), high-glucose DMEM (WISENT), and dual antibiotics (1% penicillin and streptomycin) (Beyotime). The culture environment was maintained at 37°C and 5% CO_2_.

### 2.4 Western blot analysis

The cells were lysed, after which the protein concentration was measured. The samples were then denatured and separated via SDS–PAGE. The proteins were subsequently transferred, blocked, incubated with antibodies (**Supplementary Table 1**), and ultimately detected via chemiluminescence methods. Western blot bands were quantified by ImageJ and normalized to β-actin. All work complied with established Western blot reporting standards.

### 2.5 Quantitative real-time polymerase chain reaction

RNA was extracted using the Virus DNA/RNA Extraction Kit 2.0 (Vazyme). For reverse transcription and quantitative polymerase chain reaction (qPCR), we closely followed the manufacturer’s protocols. Briefly, we used 2 × Q3 SYBR qPCR Master Mix (TOLOBIO) and ToloScript All-in-one RT EasyMix for qPCR (TOLOBIO). The relative gene expression was calculated using the 2^–ΔΔCt method, with β-actin serving as the internal reference gene. The primer sequences are detailed in **Supplementary Table 2**.

### 2.6 siRNA transfection

Once the HCC cells reached 60% confluence within a 6-well culture plate, 5 µl of siRNA was added to an enzyme-free sterile EP tube, followed by the addition of 35 µl of solution A and 5 µl of solution B for transfection, according to the CALNP™ RNAi *in vitro* (D-Nano) protocol. si-CLGN was custom-made by HanBio Shanghai (Hanhang Biological Technology Co., Ltd., Shanghai). The efficacy of transfection was confirmed by Western blot and qRT–PCR. The sequences were as follows:

si-CLGN sense: 5’-CGACAUAUGUAUACCACAATTC;
Antisense: 5’-UUGUGGUAUACAUAUGUCGTT-3’.
NC group sense: 5’-UUCUCCGAACGUGUCACGUTT-3’;
Antisense: 5’-ACGUGACACGUCGGAGAATT-3’.

### 2.7 Lentivirus transduction

In the lentiviral transfection experiment, the short hairpin RNA (shRNA) lentivirus encoding CLGN was designed and synthesized by Gemma Company (Shanghai, China). The shRNA target sequence:sh-CLGN 5’-CGACATATGTATACCACAA-3’,sh-NC 5’-TTCTCCGAACGTGCACGT-3’. The transductants were added to the culture medium supplemented with virus for 24 hours, which was followed by incubation in complete culture medium for 48 hours. The cells were screened with puromycin, and the transduction efficiency was verified by Western blot and qRT–PCR.

### 2.8 EdU cell proliferation assay

According to the instructions of the EdU Cell Proliferation Assay Kit (Beyotime), an appropriate volume of EdU working solution was added to the cell culture medium. After 3 hours of incubation, the cells were fixed, permeabilized, and stained. The fluorescence intensity and distribution were observed using an upright fluorescence microscope (Leica).

### 2.9 Cell viability/inhibition analysis

We seeded six thousand cells into each well of a 96-well plate. After 0, 24, 48, and 72 hours of culture, 10 µl of CCK-8 detection reagent (Beyotime) was added to each well (29). The absorbance was measured at a wavelength of 450 nm following the incubation period. The half-maximal inhibitory concentration (IC^50^) was calculated by generating a growth curve with the drug concentration on the horizontal axis and the cell viability or inhibition rate on the vertical axis.

### 2.10 Colony formation assay

An equivalent quantity of cells was seeded into every well of a six-well plate and subsequently cultured. Stable colonies formed after 14 days; these colonies were fixed with paraformaldehyde, subjected to crystal violet staining, and then enumerated.

### 2.11 Annexin V-FITC/PI apoptosis detection

The cells were digested in trypsin without EDTA, washed twice with PBS (1500 RPM, 5 minutes), and incubated with 5 µl of Annexin V-FITC and 10 µl of PI (15 minutes, 4°C) (30). Apoptosis was detected using a Beckman CytoFLEXLX flow cytometer.

### 2.12 Migration and invasion assays

Transwell chambers with 8-μm pores (Corning, USA) were coated with Matrigel (Corning) to evaluate the invasion ability of the cells. The migrated cells were directly fixed, stained with 0.1% crystal violet, photographed and counted under a microscope.

### 2.13 Wound healing assay

After the cells reached 90% confluence in a 6-well plate, a scratch was made across each well with a 200 µl pipette tip. Changes in the scratch width at 0 hours and 48 hours were observed by microscopy and recorded.

### 2.14 Animal experiments

BALB/c nude mice (3–4 weeks old, n=20) were purchased from GemPharmatech Co., Ltd. (Jiangsu, China). After one week of acclimatization, Hep-G2 cells (3 × 10^6^ cells) from the control (shNC) and stable knockout (shCLGN) groups were subcutaneously injected into the right armpit of each mouse. The tumor volume was measured every 5 days. Five mice from each group were randomly selected and treated with Pae for 21 consecutive days when the tumors reached 50 mm³. Researchers were blinded to group assignments during the evaluation of the results. The Institute of Health and Medicine, which is associated with the Hefei Comprehensive National Science Center, has established clearance protocols through its Institutional Animal Care and Use Committee (IACUC), as indicated by the reference number (NO. IHM-AP-2024-029).

### 2.15 Statistical analysis

All data are presented as mean ± standard deviation (SD). Statistical analyses were performed using GraphPad Prism 8.5. For comparisons between two groups, we used unpaired or paired Student’s t-tests, or Welch’s t-test when variances were unequal. For comparisons among three or more groups, we used one-way ANOVA followed by appropriate *post hoc* tests (Dunnett’s for comparison to a single control; Tukey’s for all pairwise comparisons).For categorical data, associations were assessed using the Chi-square test. For survival analysis, Kaplan-Meier curves were compared with the Log-rank test, and hazard ratios were calculated. Non-parametric alternatives (e.g., Mann-Whitney U, Kruskal-Wallis tests) were used when data did not meet normality assumptions. (*P < 0.05, **P < 0.01, ***P < 0.001.).

## 3 Results

### 3.1 ERS increases CLGN expression in HCC

**Figure 1:**
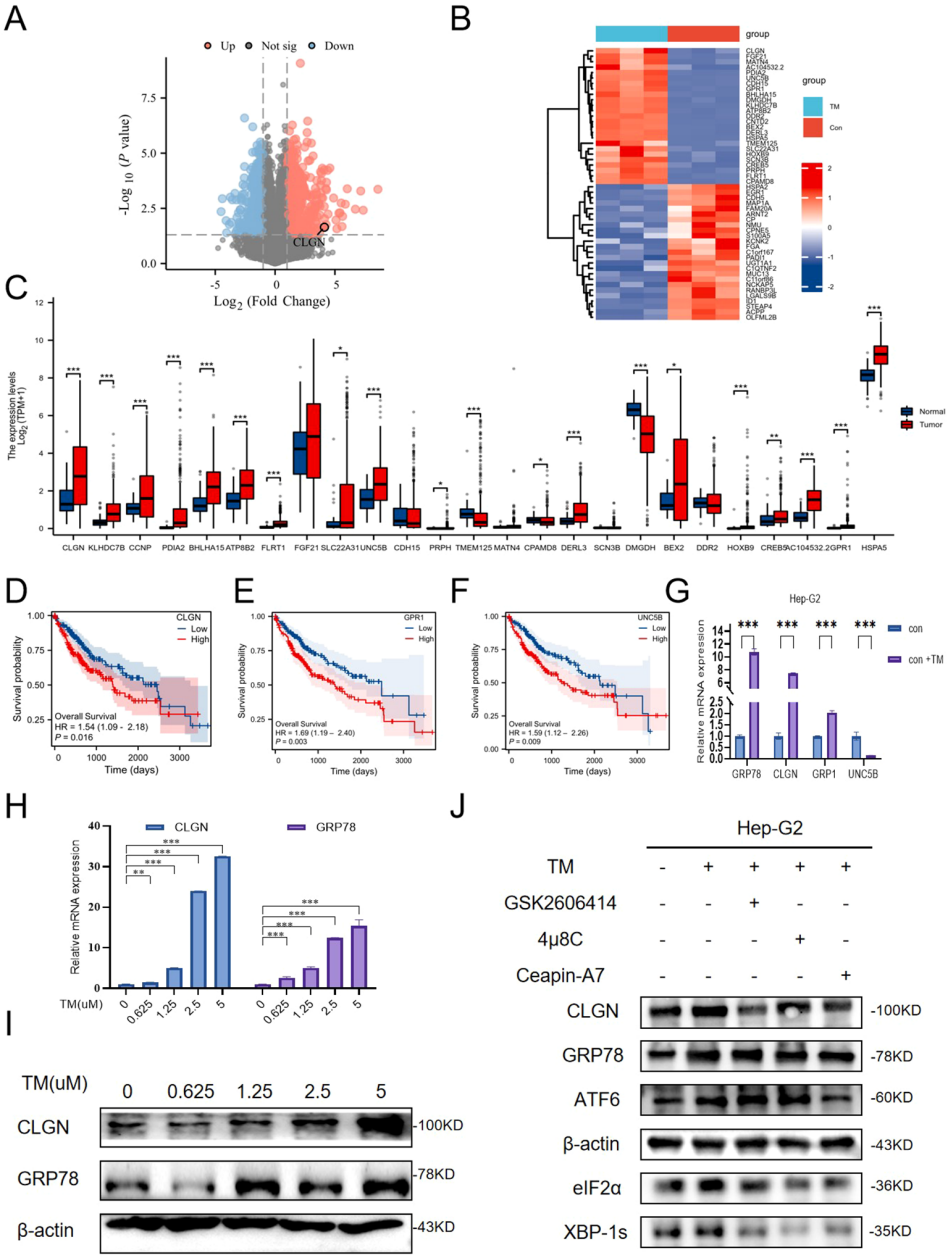
ERS upregulates CLGN expression in HCC. **(A)** Volcano plot of differentially expressed genes from mRNA sequencing of Hep-G2 cells. Red and blue dots represent significantly up- and down-regulated genes, respectively (CLGN is labeled). **(B)** Heatmap of the top 25 up- and down-regulated genes from mRNA sequencing. **(C)** Expression levels of the top 25 upregulated genes in HCC and adjacent normal tissues from the TCGA database. **(D–F)** Kaplan-Meier survival analysis of HCC patients stratified by high and low expression of *CLGN***(D)**, *GPR1***(E)**, and *UNC5B***(F)**. **(G)** qRT–PCR analysis of candidate gene expression in Hep-G2 cells treated with or without TM (unpaired Student’s t-test). **(H, I)** Dose-dependent effects of the ERS inducer TM on CLGN and GRP78 expression in Hep-G2 cells, as determined by qRT–PCR **(H)** and Western blot **(I)** (one-way ANOVA with Dunnett’s *post hoc* test). **(J)** CLGN protein expression under UPR pathway inhibition. Data are presented as mean ± SD. *P < 0.05, **P < 0.01, ***P < 0.001.

ERS-related genes with prognostic significance in HCC, we treated Hep-G2 cells with TM to induce ERS and performed transcriptome sequencing. We identified 1,062 differentially expressed genes, including 601 upregulated ones (**Figure 1A**). For subsequent analysis, we focused on the most significantly altered transcripts by selecting the top 25 upregulated and top 25 downregulated genes based on log_2_-fold change (P < 0.05) (**Figure 1B**).

We then assessed the clinical relevance of these top 25 upregulated genes using the TCGA-LIHC cohort. Seventeen of them showed significantly higher expression in tumor tissues than in adjacent non-tumor tissues (**Figure 1C**, **Supplementary Figure 1A**). Survival analysis of these 17 candidates revealed that high expression of only four genes—GPR1, HSPA5 (encoding GRP78), UNC5B, and CLGN—was significantly associated with poor overall survival (P < 0.05) (**Figures 1D–F**, **Supplementary Figure 1B**), thus narrowing our candidate pool to these four prognostic factors.

We next sought to determine which of these four genes was most robustly regulated by ERS. qRT–PCR analysis in TM-treated Hep-G2 cells showed that while the ERS marker GRP78 was, as expected, the most induced, CLGN exhibited the most pronounced upregulation among the four prognostic candidates (**Figure 1G**). In contrast, GPR1 and UNC5B showed weaker induction. This strong functional link to ERS prompted us to select CLGN for further investigation. Subsequent experiments confirmed a dose-dependent increase in CLGN mRNA and protein levels upon TM treatment (**Figures 1H, I**) and a decrease upon treatment with the ERS inhibitor 4-PBA (**Supplementary Figures 2A, B**).

Having established CLGN as an ERS-responsive gene, we next sought to delineate its regulation by individual UPR arms. Interrogation of TCGA-LIHC data revealed that CLGN expression correlated most significantly with key effectors of the PERK pathway (EIF2AK3, R = 0.362; ATF4, R = 0.344; DDIT3, R = 0.367). Weaker correlations were observed with the IRE1α (ERN1, R = 0.189; XBP1s, R = 0.297) and ATF6 (ATF6, R = 0.218) pathways (**Supplementary Figure 2C**). Furthermore, we utilized TM to promote ERS and employed PERK pathway inhibitors (GSK2606414), IRE1a pathway inhibitors (4u8c), and ATF6 pathway inhibitors (Ceapin-A7) to respectively inhibit the three pathways of UPR. qRT-qPCR and Western blot were used to detect the effects of the three pathway inhibitors on the mRNA and protein expression of CLGN. The results indicated that all three UPR branches affected the mRNA (**Supplementary Figure 2D**) and protein levels of CLGN to varying degrees (**Figure 1J**), which might be due to their synergistic regulation; however, the PERK and ATF6 pathways had more significant regulatory effects on the expression of CLGN.

In summary, through a multi-step screening process, we identify CLGN as a key prognostic target of the ERS pathway in HCC.

### 3.2 CLGN expression in HCC is correlated with adverse clinicopathological features

**Figure 2:**
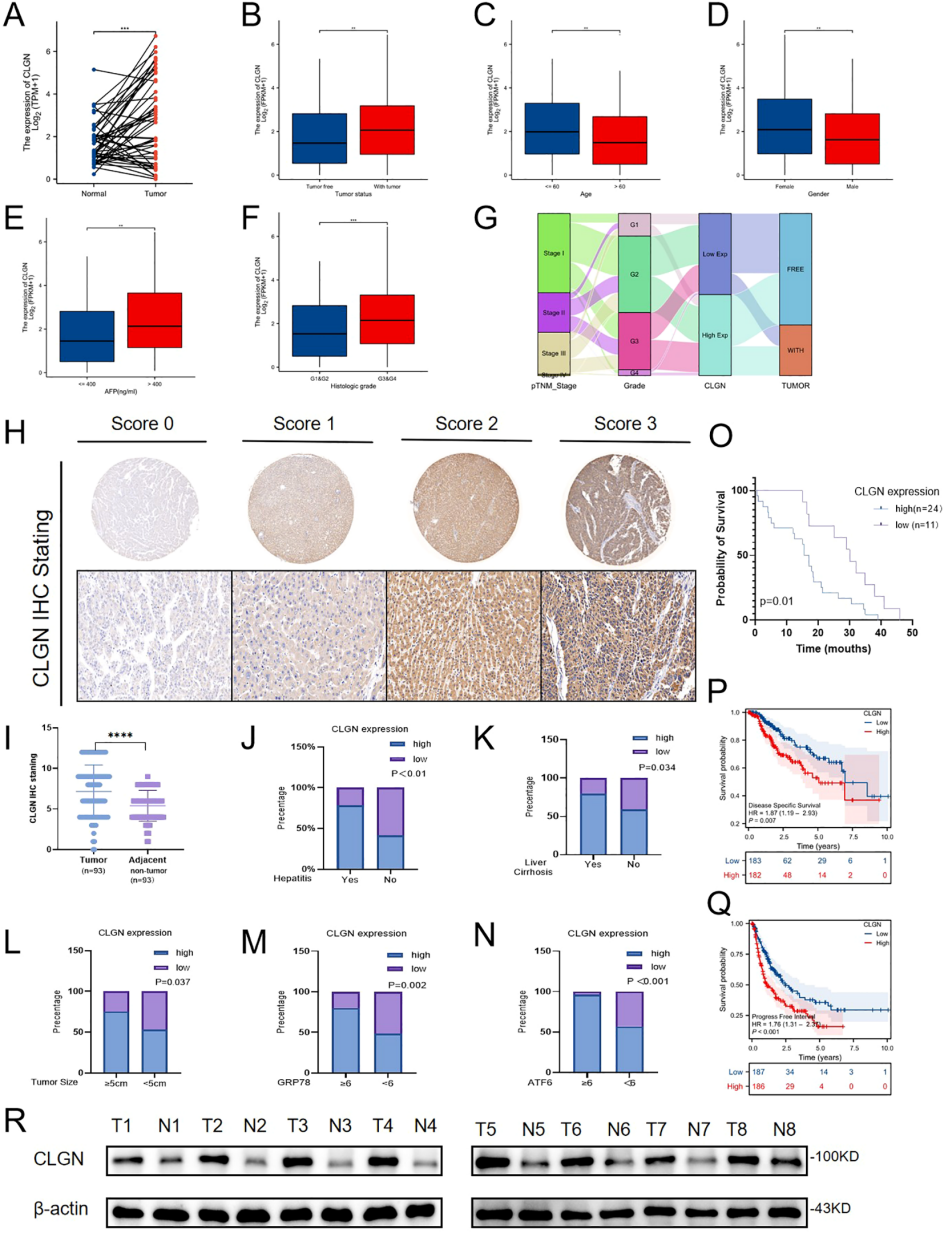
High CLGN expression correlates with aggressive clinicopathological features and poor prognosis in HCC. **(A)** CLGN mRNA expression in unpaired HCC and normal liver tissues from the TCGA-LIHC cohort (unpaired Student’s t-test). **(B–F)** Analysis of CLGN mRNA expression levels in the TCGA cohort stratified by **(B)** tumor status, **(C)** age, **(D)** sex, **(E)** serum AFP level, and **(F)** histological grade (unpaired Student’s t-test or one-way ANOVA). **(G)** Sankey diagram illustrating the flow and association between TNM stage, histological grade, CLGN expression level, and tumor status. **(H)** IHC images of CLGN staining in HCC tissues, classified into four grades (0-3) based on staining intensity. **(I)** Statistical analysis of CLGN IHC scores in HCC tissues compared with adjacent non-tumor tissues (paired Student’s t-test). **(J–L)** Analysis of CLGN IHC scores stratified by **(J)** hepatitis status, **(K)** liver cirrhosis status, and **(L)** tumor size (unpaired Student’s t-test). **(M, N)** Correlation between CLGN protein expression and the ERS markers **(M)** GRP78 and **(N)** ATF6. Patients were grouped based on the median IHC score of each ERS marker (unpaired Student’s t-test). **(O)** Kaplan-Meier analysis of overall survival based on CLGN IHC staining in our institutional cohort (n=35, Log-rank test). **(P, Q)** Kaplan-Meier survival analysis of the TCGA-LIHC cohort based on *CLGN* mRNA expression levels, showing **(P)** disease-specific survival and **(Q)** overall survival (Log-rank test). **(R)** Western blot analysis of CLGN protein expression in 8 paired fresh-frozen HCC (T) and adjacent non-tumor (N) tissues. GAPDH was used as a loading control. Data are presented as mean ± SD. *P < 0.05, **P < 0.01, ***P < 0.001.

We evaluated the relationships between CLGN expression and various clinicopathological characteristics, after which we assessed the expression of ERS markers in HCC tissues. According to the TCGA database, CLGN was highly expressed in paired and unpaired HCC tissues compared with normal tissues (**Figure 2A**, **Supplementary Figure 3A**). Its expression correlated with tumor presence (**Figure 2B**), age (**Figure 2C**), sex (**Figure 2D**), AFP level (**Figure 2E**), and histological grade (**Figure 2F**).To visualize the relationships between CLGN expression and clinicopathological features, a Sankey diagram was generated (**Figure 2G**). Higher pTNM stage, increased histological grade, and confirmed tumor presence were strongly associated with the high CLGN expression group. In contrast, early-stage and lower-grade tumors correlated more frequently with low CLGN expression. These results provide visual evidence that elevated CLGN expression is intricately linked to a more aggressive HCC phenotype.

Immunohistochemical staining for CLGN was performed on microarrays containing tumor samples and corresponding adjacent tissues from 93 HCC patients. Staining was classified into four grades (0–3) according to intensity (**Figure 2H**). The findings from the immunohistochemical analysis demonstrated that CLGN was significantly overexpressed in HCC (**Figure 2I**). Furthermore, this overexpression was correlated with hepatitis (**Figure 2J**), cirrhosis (**Figure 2K**), and tumor size (**Figure 2L**) (**Supplementary Table 3**). CLGN expression was also associated with the expression of the ERS-related proteins GRP78 and ATF6 (**Figures 2M, N**). To preliminarily assess the clinical relevance of CLGN protein expression, we performed IHC on 35 paired HCC specimens and conducted survival analysis. Although the cohort size is limited, our results revealed a trend where high CLGN expression was associated with poor overall survival (**Figure 2O**). Importantly, this finding is strongly supported by an independent analysis of the large TCGA-LIHC cohort, which confirmed at the mRNA level that high CLGN expression correlates with significantly shorter overall survival and disease-specific survival (**Figures 2P, Q**). This consistent evidence across two independent datasets strongly suggests that CLGN is a robust prognostic indicator in HCC.

Furthermore, to validate the expression difference at the protein level, we examined 8 pairs of fresh HCC tissues alongside adjacent nontumor tissues by Western blot. The results consistently demonstrated significantly increased CLGN expression in HCC tissues (**Figure 2R**).

### 3.3 CLGN promotes HCC cell proliferation *in vitro*

**Figure 3:**
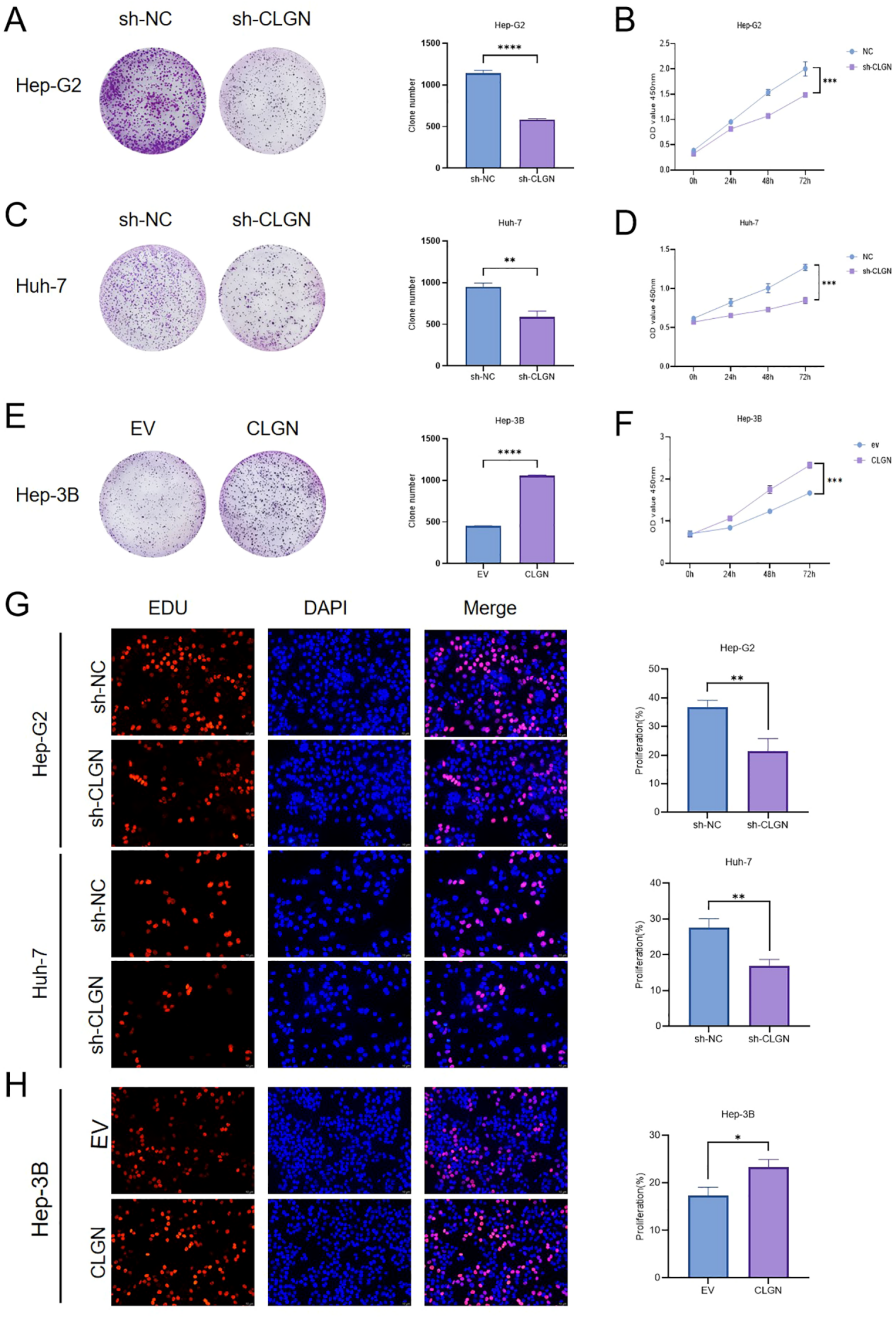
CLGN promotes HCC cell proliferation *in vitro*. **(A, B)** Proliferation of Hep-G2 cells with stable CLGN knockdown was assessed by **(A)** colony formation assay and **(B)** CCK-8 assay. **(C, D)** Proliferation of Huh-7 cells with stable CLGN knockdown was assessed by **(C)** colony formation assay and **(D)** CCK-8 assay. **(E, F)** Proliferation of Hep-3B cells with stable CLGN overexpression was assessed by **(E)** colony formation assay and **(F)** CCK-8 assay. **(G)** Proliferation of CLGN-knockdown Hep-G2 and Huh-7 cells was assessed by EdU assay. Scale bar, 50 μm. **(H)** Proliferation of CLGN-overexpressing Hep-3B cells was assessed by EdU assay. Scale bar, 50 μm. Data are presented as mean ± SD. *P < 0.05, **P < 0.01, ***P < 0.001 (Student’s t-test or one-way ANOVA).

To explore the role of CLGN in HCC, we verified CLGN expression in different HCC cell lines via Western blot and qRT–PCR (**Supplementary Figures 3B, C**). In addition to the Hep-G2 cells used for sequencing, we selected the Huh-7 cell line, which has relatively high CLGN expression. Effective siRNA fragments were screened using Western blot (**Supplementary Figures 3D, E**). The CLGN gene was targeted for knockdown in two distinct cell lines using lentiviral technology, and the effectiveness of this approach was confirmed through Western blot and qRT–PCR (**Supplementary Figures 3F, G**, **Supplementary Figure 3I**). CLGN was also overexpressed in Hep-3B cells, which have relatively low endogenous CLGN expression (**Supplementary Figures 3H, J**). CLGN knockdown significantly inhibited the *in vitro* proliferation of Hep-G2 and Huh-7 cells, as shown by colony formation, CCK-8, and EdU assays (**Figures 3A–D, G**). Conversely, CLGN overexpression promoted Hep-3B cell proliferation (**Figures 3E–F, H**).

### 3.4 CLGN promotes HCC cell invasion and migration and suppresses apoptosis *in vitro*

**Figure 4:**
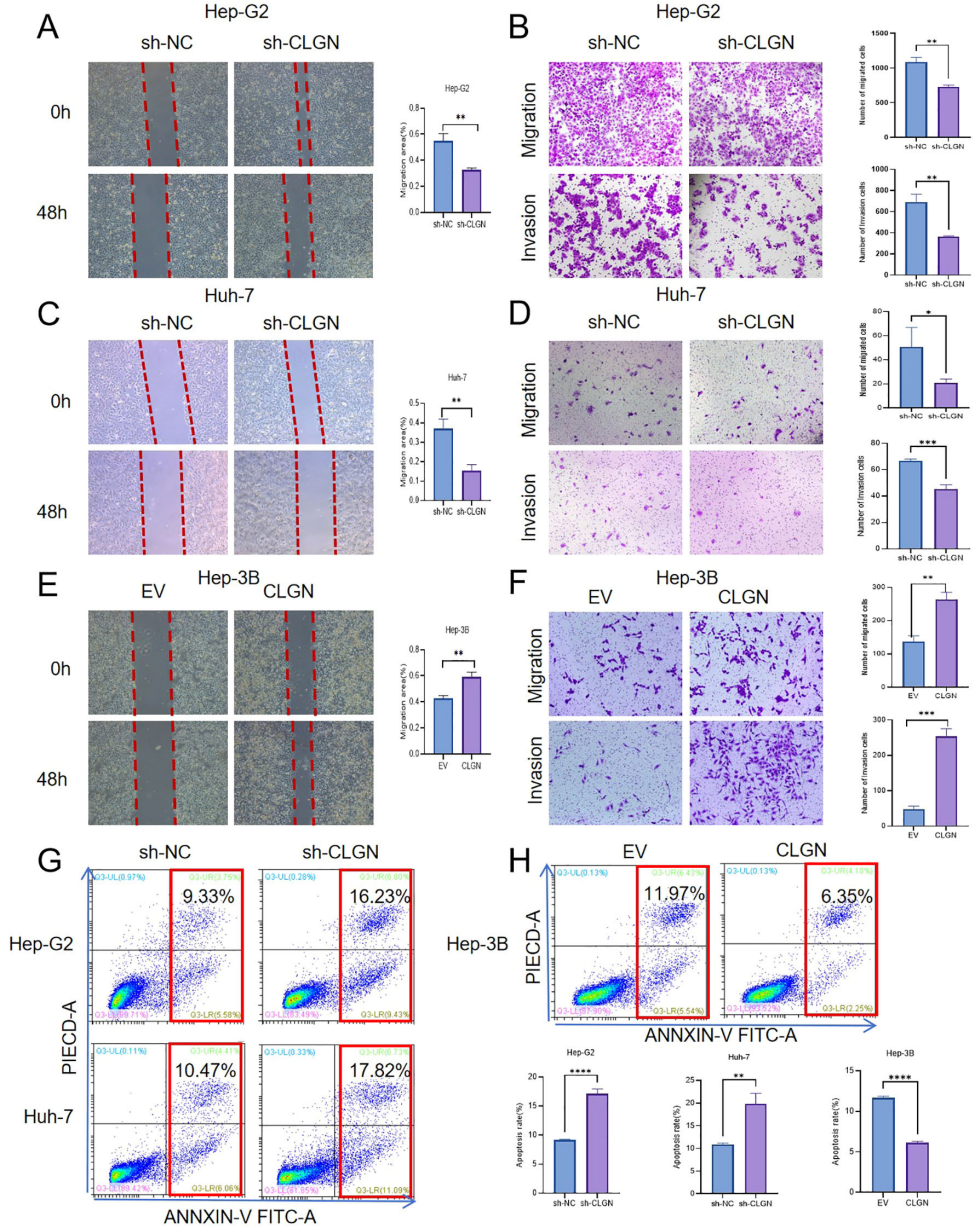
CLGN promotes invasion, migration, and suppresses apoptosis in HCC cells *in vitro*. **(A, B)** Effects of CLGN knockdown in Hep-G2 cells on **(A)** wound healing migration and **(B)** Transwell invasion. **(C, D)** Effects of CLGN knockdown in Huh-7 cells on **(C)** wound healing migration and **(D)** Transwell invasion. **(E, F)** Effects of CLGN overexpression in Hep-3B cells on **(E)** wound healing migration and **(F)** Transwell invasion. **(G)** Apoptosis analysis by flow cytometry in CLGN-knockdown Hep-G2 and Huh-7 cells. **(H)** Apoptosis analysis by flow cytometry in CLGN-overexpressing Hep-3B cells. Data are presented as mean ± SD. *P < 0.05, **P < 0.01, ***P < 0.001 (Student’s t-test or one-way ANOVA).

We next investigated the impact of CLGN on metastatic potential and apoptosis. Wound healing, Transwell, and Annexin V-FITC/PI assays revealed that CLGN knockdown in Hep-G2 and Huh-7 cells significantly attenuated their migratory and invasive capacities and concurrently promoted apoptosis (**Figures 4A–D, G**). Conversely, CLGN overexpression in Hep-3B cells enhanced invasion and migration while inhibiting apoptosis (**Figures 4E, F, H**).

### 3.5 CLGN knockdown sensitizes HCC cells to paeonol by attenuating ERS

**Figure 5:**
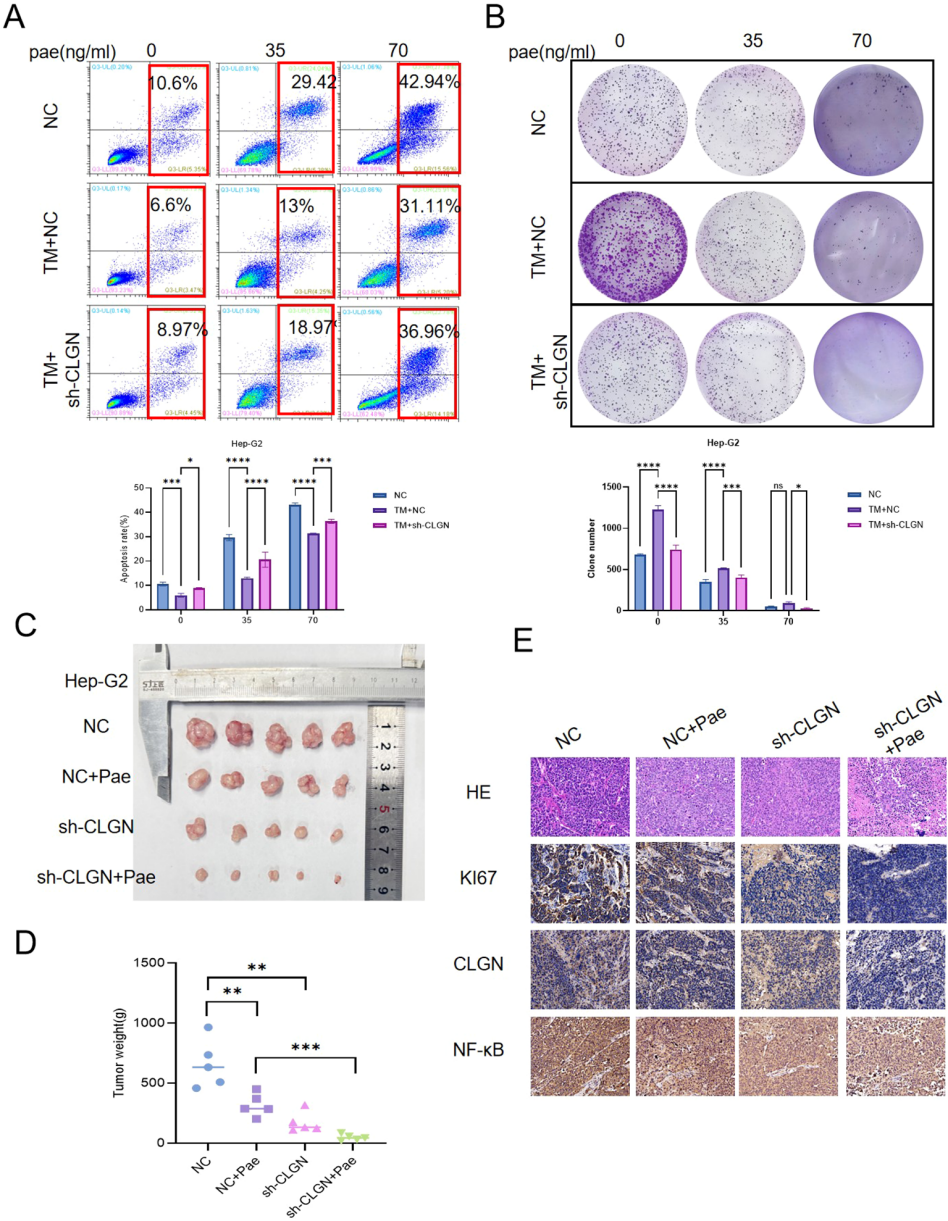
CLGN knockdown enhances the anti-tumor efficacy of Pae by modulating ERS. **(A, B)** Hep-G2 control and CLGN-knockdown cells were treated with TM and/or Pae, followed by analysis of **(A)** apoptosis via flow cytometry and **(B)** clonogenic survival. **(C)** Representative images of resected tumors from the xenograft mouse model under different treatment conditions. **(D)** Tumor weights from each treatment group at the endpoint. **(E)** IHC analysis of Ki67, CLGN, and NF-κB expression in xenograft tumor tissues. Data are presented as mean ± SD. *P < 0.05, **P < 0.01, ***P < 0.001 (B, D: one-way ANOVA with Tukey’s *post hoc* test; A: two-way ANOVA).

Given the role of CLGN in ERS, we asked whether targeting CLGN could enhance the efficacy of the anti-HCC agent Pae. The IC_50_ value of pae in HepG2 cells was determined to be 68.98 ng/mL (**Supplementary Figure 3K**). We observed that TM-induced ERS promoted cell proliferation, suppressed apoptosis, and induced resistance to Pae. Notably, CLGN knockdown reversed these pro-tumorigenic effects and restored Pae sensitivity. The combination of sh-CELGN and Pae most effectively suppressed proliferation and induced apoptosis under ERS conditions (**Figures 5A, B**).

To translate these findings *in vivo*, we employed a xenograft mouse model. While Pae monotherapy or sh-CLGN alone moderately inhibited tumor growth, the combination of sh-CLGN and Pae resulted in the most potent suppression of tumor growth (**Figures 5C, D**). Immunohistochemical analysis of the xenograft tumors further confirmed the enhanced anti-tumor effect of the combination therapy, as evidenced by the lowest expression levels of the proliferation marker Ki67 and the pro-survival protein NF-κB in this group (**Figure 5E**).

### 3.6 CLGN suppresses apoptosis in HCC via the NF-κB signaling pathway

**Figure 6:**
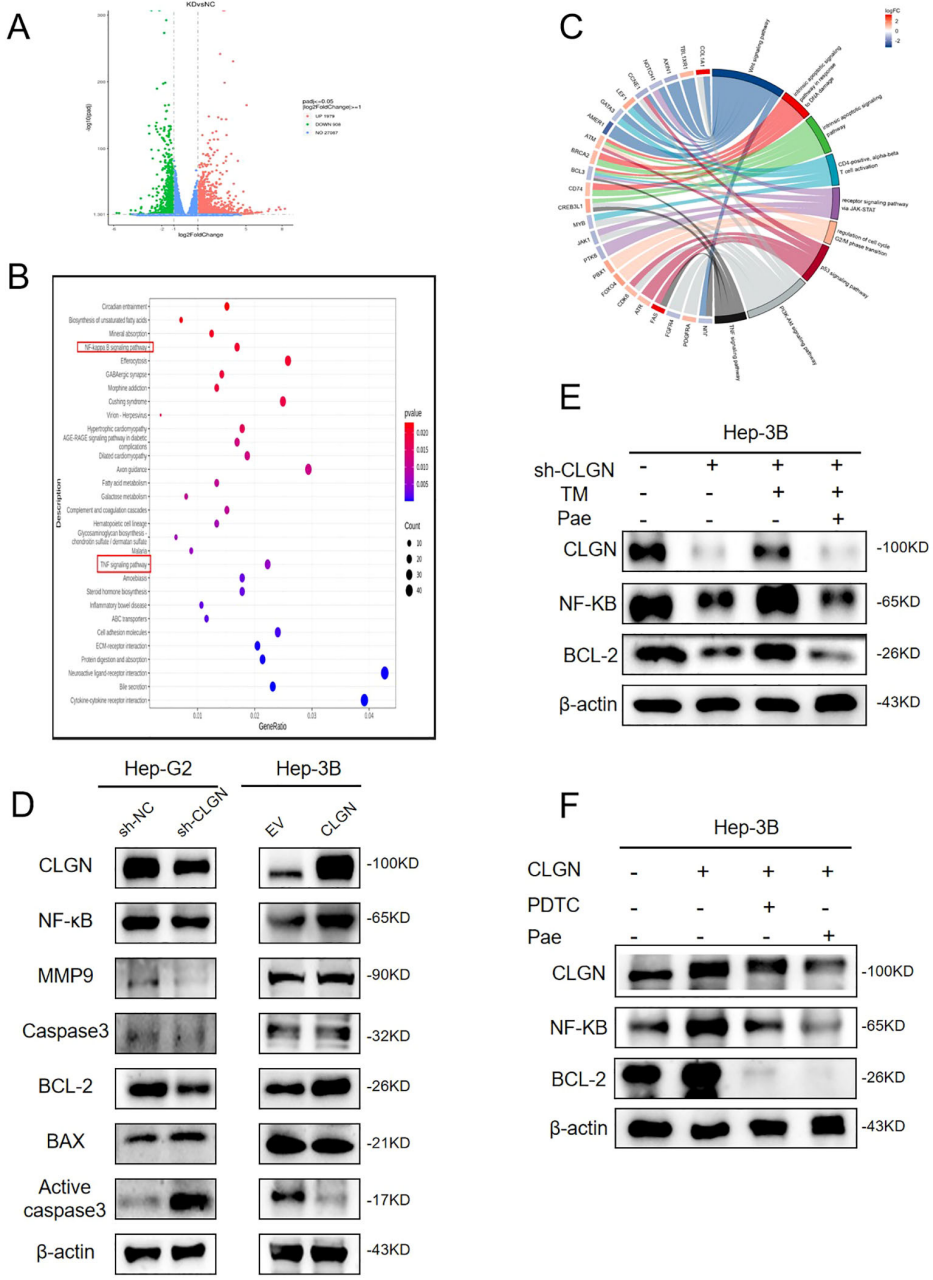
CLGN suppresses apoptosis through activation of the NF-κB pathway. **(A)** Volcano plot of DEGs from RNA sequencing of control versus CLGN-knockdown Hep-G2 cells. **(B)** Chord plot illustrating the results of combined GO/KEGG and logFC enrichment analysis for the identified DEGs. **(C)** Bar graph of the most significantly enriched KEGG pathways. **(D)** Western blot analysis of key NF-κB pathway proteins in Hep-G2 with CLGN knockdown and Hep-3B cells with CLGN overexpression. **(E)** Western blot analysis of CLGN, NF-κB, and Bcl-2 expression in control and CLGN-knockdown Hep-G2 cells treated with TM or TM+Pae. **(F)** Western blot analysis of CLGN, NF-κB, and Bcl-2 expression in vector-control and CLGN-overexpressing Hep-3B cells treated with the NF-κB inhibitor PDTC or Pae. GAPDH was used as a loading control for all Western blot analyses.

To elucidate the mechanism by which CLGN promotes HCC progression, we performed transcriptome sequencing of control and CLGN-knockdown cells. This analysis identified 2887 differentially expressed genes (DEGs), comprising 1979 upregulated and 908 downregulated genes (**Figure 6A**, **Supplementary Figure 4A**). Gene Ontology (GO) and Kyoto Encyclopedia of Genes and Genomes (KEGG) enrichment analyses revealed that these differentially expressed genes (DEGs) were significantly enriched in the TNF and NF-κB signaling pathways (**Figures 6B, C**). Western blot validation confirmed that CLGN knockdown modulated the expression of key effector proteins within the NF-κB pathway (**Figure 6D**), suggesting that CLGN exerts its anti-apoptotic effect, at least in part, through NF-κB activation.

We further probed this functional axis under ERS conditions. As shown in **Figure 6E**, TM-induced ERS upregulated both CLGN and NF-κB expression and suppressed apoptosis in Hep-G2 cells. This effect was counteracted by the combined treatment of sh-CLGN and Pae, which robustly induced apoptosis. Conversely, in CLGN-overexpressing Hep-3B cells, treatment with either the NF-κB inhibitor PDTC or Pae reduced NF-κB activation and decreased the expression of the anti-apoptotic protein Bcl-2 (**Figure 6F**). Intriguingly, Pae treatment also downregulated CLGN expression itself, implying that its anti-tumor effect may be mediated partly through suppressing the CLGN/NF-κB axis.

## 4 Discussion

The endoplasmic reticulum (ER) is a pivotal organelle responsible for protein synthesis, folding, and modification (18, 31). arises when ER homeostasis is disrupted, leading to the accumulation of unfolded or misfolded proteins (32). Accumulating evidence underscores the critical role of ERS in tumorigenesis and cancer progression (33, 34). Notably, ERS not only empowers tumor cells to evade immune surveillance (35, 36) but also orchestrates key oncogenic processes (37), including proliferation (14), apoptosis evasion, and drug resistance (38). However, the specific molecular mechanisms driving these effects in HCC remain incompletely elucidated.

Previous bioinformatics studies, such as those by CHENG et al. utilizing machine learning algorithms, have identified CLGN as a potential ERS-related gene (39). Furthermore, CLGN upregulation, regulated by miR-194-3p, has been associated with poor prognosis in HCC (40). Building upon this foundation, our study provides direct functional evidence. By constructing an ERS model and employing transcriptome sequencing, we identified CLGN as a key downstream target. We confirmed the strong correlation between CLGN overexpression, ERS activation, and unfavorable patient outcomes. Through *in vitro* and *in vivo* experiments, we demonstrated that CLGN promotes HCC cell proliferation, migration, and invasion while suppressing apoptosis.

A key finding of our research is the potential mechanistic link between CLGN and apoptosis resistance. Our data suggest that CLGN may exert its anti-apoptotic effect, at least in part, through modulating the NF-κB signaling pathway. While this specific role of CLGN in HCC has not been previously reported, our findings provide a plausible mechanistic perspective on how ERS promotes HCC malignancy. Further investigation is warranted to fully delineate this pathway.

The clinical management of HCC is often hampered by the side effects, including cardiotoxicity, of many chemotherapeutic agents (41, 42). Paeonol (Pae), a natural compound with documented cardioprotective properties, presents a promising therapeutic candidate. However, its efficacy as a monotherapy is limited. Our study suggests a possible explanation: the ERS microenvironment may foster resistance to Pae, potentially through a CLGN-mediated mechanism. It is noteworthy that a residual induction of CLGN was observed in knockdown cells upon TM treatment (**Figure 5E**), indicating that potent ERS inducers can partially overcome the knockdown efficiency. This finding underscores the strength of the ERS-CLGN axis and suggests that complete abrogation of this pathway might require a combination of genetic and pharmacological inhibition. In support of this, we found that CLGN knockdown sensitizes HCC cells to Pae-induced apoptosis *in vitro*, and our Western blot analysis points to a potential “CLGN/NF-κB/Bcl-2” regulatory axis. This insight could explain the constrained effectiveness of Pae alone and provides a rationale for exploring combination therapy.

Based on these insights, we propose a novel strategy: combining CLGN inhibition with Pae treatment to potentially overcome apoptosis resistance in HCC cells under ERS conditions. Our findings establish a preliminary theoretical foundation for this approach.

Despite these findings, our study has limitations. The exploration of CLGN’s function is primarily focused on the NF-κB pathway, yet transcriptome data imply it may influence other processes. Furthermore, the precise molecular interactions by which CLGN regulates NF-κB remain to be fully elucidated. Future studies should employ multi-omics integrated analysis and detailed molecular experiments to systematically map the broader signaling network regulated by CLGN and Pae.

In conclusion, our research suggests a novel ERS-driven mechanism in HCC that may involve CLGN. These findings position CLGN as a potential therapeutic target and indicate that its inhibition might enhance the efficacy of Pae. The combination strategy offers a promising direction for future research into HCC treatment (43).

## 5 Conclusions

In summary, our study provides evidence supporting a model in which CLGN, upregulated by endoplasmic reticulum stress (ERS), contributes to hepatocellular carcinoma (HCC) progression and drug resistance, potentially via the NF-κB pathway. Our key findings indicate that (1): ERS upregulates CLGN expression, which is associated with enhanced HCC cell proliferation, migration, and anti-apoptotic activity (2); Elevated CLGN expression correlates with aggressive clinicopathological features and poor patient prognosis (3); The natural compound Paeonol (Pae) appears to counteract CLGN-mediated resistance, potentially by suppressing the CLGN/NF-κB axis, thereby restoring apoptosis sensitivity and inhibiting tumor growth in preclinical models. However, this study has limitations. The mechanistic insights are primarily derived from a limited set of cell lines and focus predominantly on the NF-κB pathway, despite transcriptomic data suggesting the involvement of other signaling networks. While preclinical models (xenografts) validate this strategy, long-term pharmacological and clinical studies are essential to assess safety and efficacy. Thus, despite its mechanistic promise, CLGN/Pae-based therapy remains distant from clinical application and requires further optimization.

## Data Availability

All the data generated or analyzed during the current study are included in this publication and its Supplementary Information files. The datasets supporting this study are available from the corresponding author upon reasonable request. The GEO accession number for the CLGN-knockdown mRNA sequencing data is GSE295052. The RNA sequencing data of TM-treated and untreated HepG2 cells are available under GEO accession number GSE208391.
