# Supplementary figures and images for: Targeting endoplasmic reticulum stress-induced CLGN resensitizes hepatocellular carcinoma to apoptosis: paeonol synergistically enhances efficacy by dual inhibition of CLGN and NF-κB
Source: Front Oncol. 2025 Nov 28;15:1709962. doi: 10.3389/fonc.2025.1709962 (PMC12698408; doi:10.3389/fonc.2025.1709962)

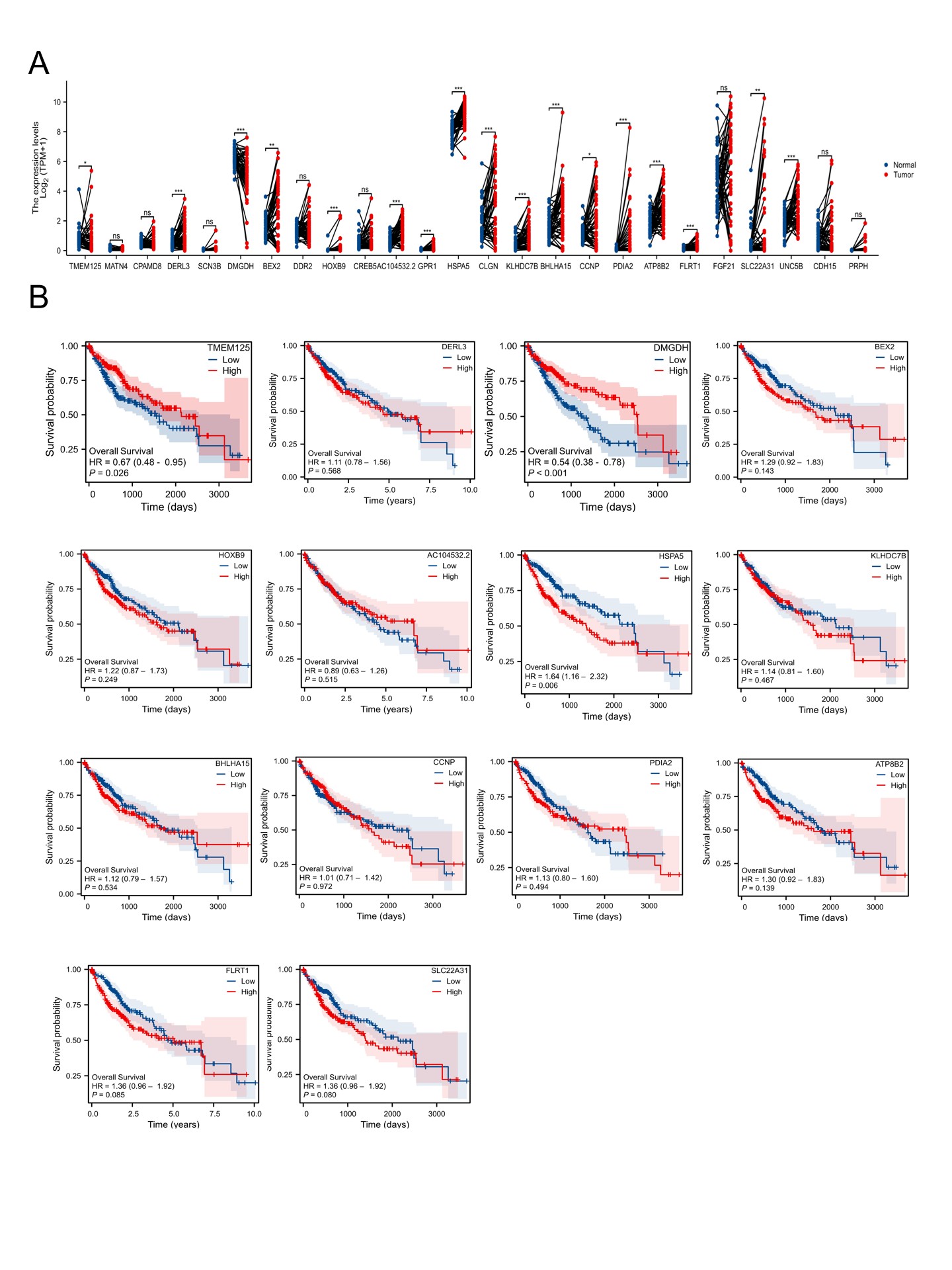

Supplement: Supplementary file 1 [file Image1.jpeg]

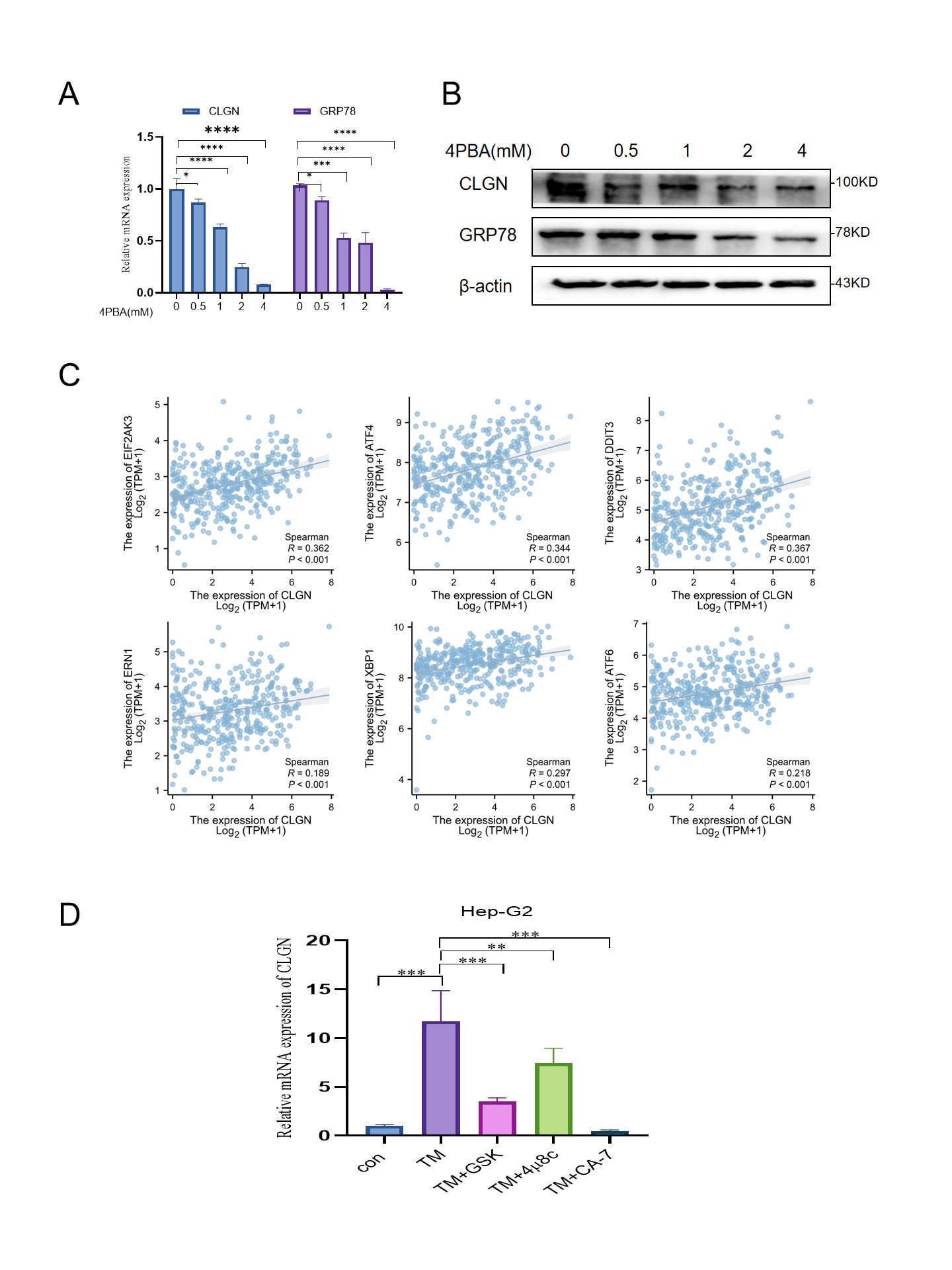

Supplement: Supplementary file 2 [file Image2.jpeg]

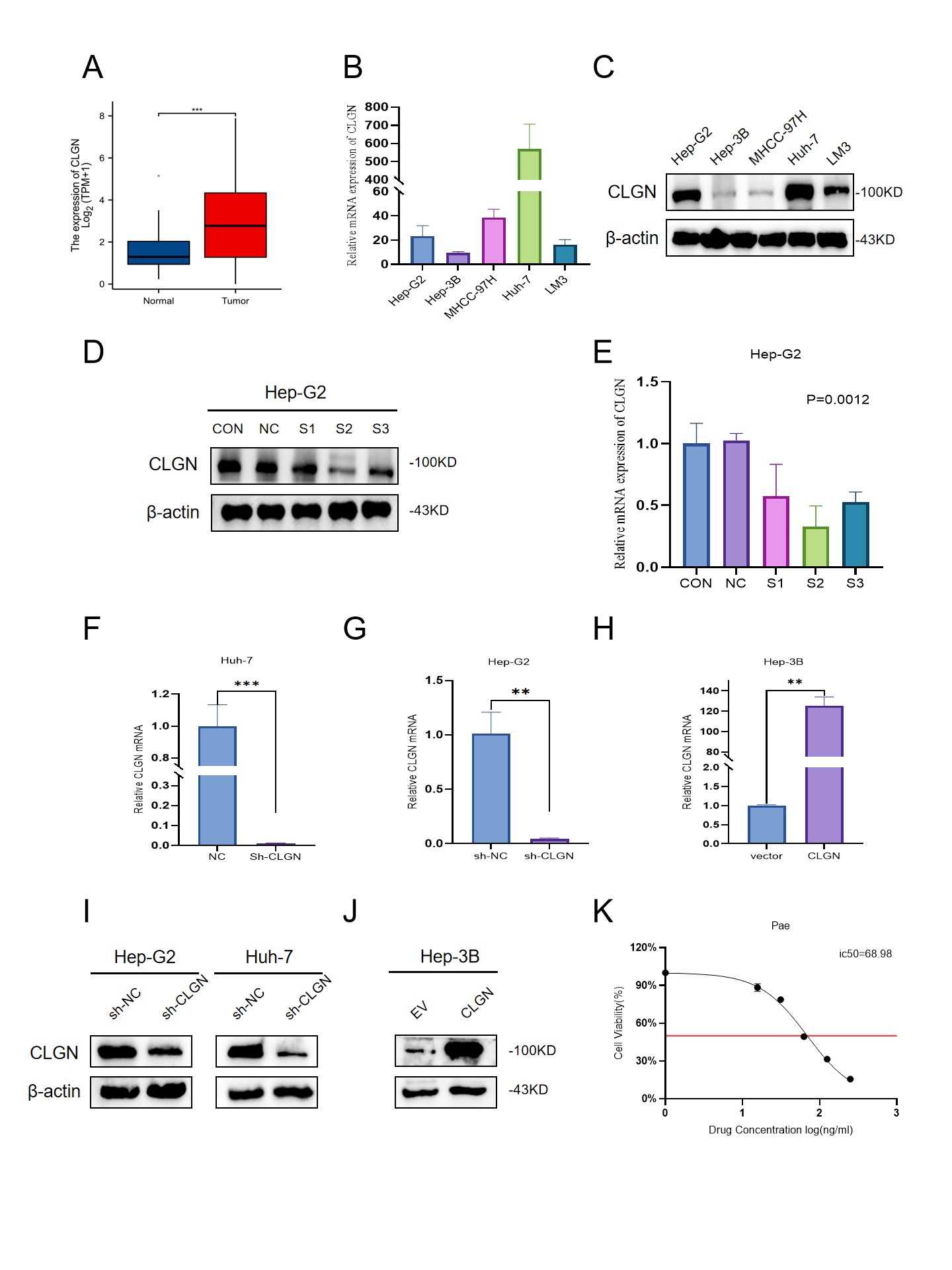

Supplement: Supplementary file 3 [file Image3.jpeg]

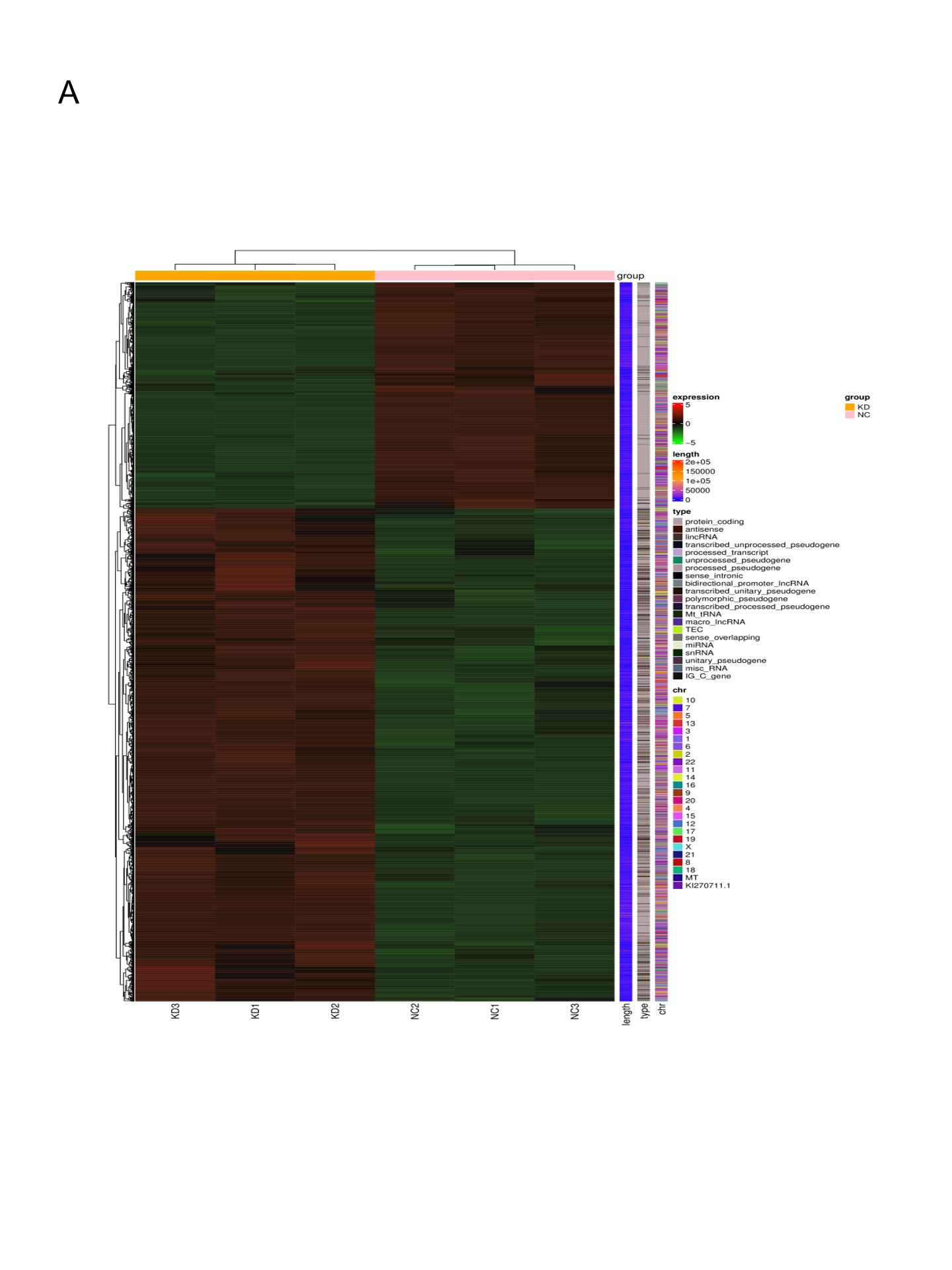

Supplement: Supplementary file 4 [file Image4.jpeg]
