# Supplementary material for: Targeting endoplasmic reticulum stress-induced CLGN resensitizes hepatocellular carcinoma to apoptosis: paeonol synergistically enhances efficacy by dual inhibition of CLGN and NF-κB
Source: Front Oncol. 2025 Nov 28;15:1709962. doi: 10.3389/fonc.2025.1709962 (PMC12698408; doi:10.3389/fonc.2025.1709962)
Supplement: Supplementary file 5 [file Table1.docx]

| **Antibody** | **Antibody dilution ratio (in Western blot)** | **Company** |
| --- | --- | --- |
| CLGN | 1:1000 | BOSTER |
| GRP78 | 1:5000 | proteintech |
| NF-KB | 1:1000 | CST |
| MMP9 | 1:1000 | ABclonal |
| Caspase-3 | 1:5000 | HUBIO |
| BCL-2 | 1:1000 | Affinity |
| BAX | 1:1000 | CST |
| Active caspase3 | 1:1000 | HUBIO |
| β-actin | 1:50000 | proteintech |
| XBP-1s | 1:1000 | CST |
| eIF2α | 1:2000 | Affinity |
| ATF6 | 1:1000 | abclonal |
