# Supplementary material for: Targeting endoplasmic reticulum stress-induced CLGN resensitizes hepatocellular carcinoma to apoptosis: paeonol synergistically enhances efficacy by dual inhibition of CLGN and NF-κB
Source: Front Oncol. 2025 Nov 28;15:1709962. doi: 10.3389/fonc.2025.1709962 (PMC12698408; doi:10.3389/fonc.2025.1709962)
Supplement: Supplementary file 6 [file Table2.docx]

**Table S2. Primer sequences for RT-qPCR**

| Target gene | Forward (5’-3’) | Reverse (5’-3’) |
| --- | --- | --- |
| CLGN | GATCCTTCTGCCGTCAAACC | ATCTGAGGTGCCTCCATTC |
| β-actin | CCCAGCCATGTACGTTGCTA | TCACCGGAGTCCATCACGAT |
| HSPA5 | CCGAGAACACGGTCTTTG AC | CTTTGTTTGCCCACCTCCAA |
| UNC5B | CAACTTCCTGCTCACCATCG | TTC ACGTAGACGATG ACGGT |
| GRP1 | GGAGAGCAAAACGACTCAGAG | CCCCAATGACGGTCTTAT TTA GG |
| PERK | ACGATGAGACAGAGTTGCGAC | ATCCAAGGCAGCAATTCTCCC |
| ATF6 | TCCTCGGTCAGTGGACTCTTA | CTTGGGCTGAATTGAAGGTTTTG |
| IREla | CACAGTGACGCTTCCTGAAAC | GCCATCATTAGGATCTGGGAGA |
