# Supplementary material for: Targeting endoplasmic reticulum stress-induced CLGN resensitizes hepatocellular carcinoma to apoptosis: paeonol synergistically enhances efficacy by dual inhibition of CLGN and NF-κB
Source: Front Oncol. 2025 Nov 28;15:1709962. doi: 10.3389/fonc.2025.1709962 (PMC12698408; doi:10.3389/fonc.2025.1709962)
Supplement: Supplementary file 7 [file Table3.docx]

| Characteristics | Low expression  of CLGN | High expression  of CLGN | P value |
| --- | --- | --- | --- |
| n | 29 | 64 |  |
| Gender, n (%) |  |  | 0.668 |
| Male | 21 (22.6%) | 49 (52.7%) |  |
| Female | 8 (8.6%) | 15 (16.1%) |  |
| Age, n (%) |  |  | 0.527 |
| ＜60 | 18 (19.4%) | 44 (47.3%) |  |
| ≥60 | 11 (11.8%) | 20 (21.5%) |  |
| Hepatitis, n (%) |  |  | < 0.001 |
| No | 14 (15.1%) | 10 (10.8%) |  |
| Yes | 15 (16.1%) | 54 (58.1%) |  |
| Liver Cirrhosis, n (%) |  |  | 0.034 |
| No | 20 (21.5%) | 29 (31.2%) |  |
| Yes | 9 (9.7%) | 35 (37.6%) |  |
| AFP, n (%) |  |  | 0.255 |
| ＜20 | 14 (15.1%) | 21 (22.6%) |  |
| 20～400 | 5 (5.4%) | 20 (21.5%) |  |
| ≥400 | 10 (10.8%) | 23 (24.7%) |  |
| Stage, n (%) |  |  | 0.918 |
| Ⅰ～Ⅱ | 21 (22.6%) | 47 (50.5%) |  |
| Ⅲ～Ⅳ | 8 (8.6%) | 17 (18.3%) |  |
| Tumor Size(cm), n (%) |  |  | 0.037 |
| ＜5 | 13 (14%) | 15 (16.1%) |  |
| ≥5 | 16 (17.2%) | 49 (52.7%) |  |
| Degree of Differentiation, n (%) |  |  | 0.586 |
| Low | 3 (3.2%) | 12 (12.9%) |  |
| Medium | 15 (16.1%) | 31 (33.3%) |  |
| High | 11 (11.8%) | 21 (22.6%) |  |
| OS(month), n (%) |  |  | 0.010 |
| ＜12 | 0 (0%) | 7 (20%) |  |
| 12～24 | 3 (8.6%) | 12 (34.3%) |  |
| ≥24 | 8 (22.9%) | 5 (14.3%) |  |
| GRP78, n (%) |  |  | 0.002 |
| Low | 17 (18.3%) | 16 (17.2%) |  |
| High | 12 (12.9%) | 48 (51.6%) |  |
| ATF6, n (%) |  |  | < 0.001 |
| Low | 28 (30.1%) | 37 (39.8%) |  |
| High | 1 (1.1%) | 27 (29%) |  |
| PERK, n (%) |  |  | 0.410 |
| Low | 29 (31.2%) | 60 (64.5%) |  |
| High | 0 (0%) | 4 (4.3%) |  |
| IRE1, n (%) |  |  | 0.407 |
| Low | 27 (29%) | 54 (58.1%) |  |
| High | 2 (2.2%) | 10 (10.8%) |  |
